# On the State of the Circulation in the Lungs

**Published:** 1831

**Authors:** 


### On the State of the Circulation in the Lungs. By Dr. Malden, of Worcester.

In our Midland contemporary of last quarter, Dr. Malden has addressed a letter to Dr. Hastings on the above subject, in which erudition and ingenuity are conspicuous.

Passing over some appropriate quotations from the fathers of physic, respecting the age at which phthisis is most likely to commence—the causes which gave it birth—and the symptoms which notify its approach, the author institutes a comparison between the symptoms of the congestive form of acute pneumonia, and those of incipient phthisis.

"It frequently happens (he observes) that, at the very onset of *inflammation of the lungs*, the pulse is small, irregular, and oppressed, yet very frequent, the patient at the same time complaining of *great weakness* and oppression; yet there are present, a load at the breast, difficulty of breathing, great anxiety and heat felt about the precordia. This sudden prostration of strength and pulse cannot depend upon real exhaustion, which the duration of the disease for a few hours could not produce. The debility, and all the other symptoms, depend upon the unequal distribution of the blood, the great accumulation of it in the lungs, the impeded function of these organs, and the imperfect supply of blood to the systemic side of the heart. 'In such cases, letting of blood is so far from weakening, that it really raises the powers of nature.'*

In some very violent cases, 'where both the lobes of the lungs are greatly inflamed and *obstructed*, an immediate and excessive weakness comes on, with an inexpressible anxiety and oppression of the chest, a very *small, weak, trembling pulse*, coldness of the extremities, clammy coldish sweats, the eyes staring, the face bloated, and almost livid, &c.'†

In this extreme and dreadful case, the blood is so accumulated in the lungs, that the aorta and its branches do not receive enough to carry on the common offices of life.

'Dissections have shewn this to be the case. The lungs have been found quite *stuffed* up with concreted blood, red, hard, and, as it were, *fleshy*, or rather of the colour and consistence of *liver*, and so heavy, that any part of them cut off, sank in water.'‡ Nothing but *immediate* and *large* bleedings can save such a deplorable case as this.

Now, the question I propose is this,—is there any analogy between the insidious symptoms of incipient phthisis and such cases as the foregoing? I think there is, as much as can be expected between acute and chronic disease of the same kind.

Phthisis usually begins with a dry cough, so slight and inconsiderable, that little or no notice is taken of it, till its *continuance* and *gradual increase* begin to make it regarded. If, at this early period, the respiration be attended to, it will *uniformly* be found to be *quicker* and *shorter* than natural. This often does not attract attention, from the circumstance of this *quick* breathing not being *laborious* when the patient is at rest; but as it readily becomes so

---

* "See Huxham on the Peripneumony, pp. 182-3."

† "Ditto ibidem.

‡ "Huxam as before."

upon slight exertion, this latter circumstance is generally noticed much earlier than the former. This state of things is generally attended with, or soon followed by, a quick, hard and small, *sometimes extremely* small, pulse, constantly above ninety, languor, lassitude, and general weakness; there are occasional, sometimes periodical, burnings of the hands and feet, but, for the most part, the extremities receive a narrow stream of blood, and are easily chilled. *A spitting of blood* has sometimes been the first symptom; this alone would not be a proof of imminent phthisis, but, conjoined with the above enumerated symptoms, it must be considered a very unfavourable sign. On the whole, *short and quick respiration*, with a *hurried small pulse*, are the two most dangerous symptoms in a suspected phthisis; and persons have died in a state of great emaciation, and their lungs, upon dissection, have been found both tuberculated and hepatized, who, when living, had no other symptoms than these two, conjoined with a hacking dry cough.

Is there not, then, acute and chronic pneumonia? and is not a congested state of the vessels of the lungs common to both? and lastly, in aggravated cases, may not death ensue from this congested state alone, or its consequences, in both forms of the disease?"

The author then makes allusion to the subject of "small bleedings in hæmoptysis," on which Dr. Cheyne has written a paper in the Dublin Transactions, already noticed in this Journal. Dr. Malden quotes some passages from Sir John Pringle, to shew that that distinguished physician had completely anticipated Dr. Cheyne in his observations, and seems not a little surprised that Dr. C. should have proposed his plan of small bleedings as *something new*, after what Sir J. Pringle had written on the same subject. Such a coincidence, however, between two practical physicians, on a point of mere personal experience, will appear much less suprising than the identity of doctrine between Huxham and Barry, as pointed out by our correspondent, Philalethes, in our last number. It is not, indeed, to be expected that medical men, immersed in the toil of practice, can wade through the records of antiquity, or even the ponderous tomes of modern works, to ascertain whether or not their practical observations have been anticipated by their ancestors. Good springs out of evil, as well as evil out of good. Many a valuable fact would remain *inoperative*, because not reiterated, if physicians were aware that it had been previously published. Thus, it is probable Dr. Barry would never have instituted a series of experiments, to prove what Huxham had long ago asserted, had he been aware of Huxham's observations, and, consequently, unstimulated by the desire and the credit of novelty and discovery. So of Pringle and Cheyne. Not one medical practitioner in one hundred ever turns over a single page of Pringle during his whole life. Pringle's recommendation, therefore, of small bleedings in hæmoptysis (a practice not new even with Pringle) might have remained unnoticed and inoperative, had not a modern physician of talents and experience revived the practice. We shall, however, give Pringle's words.

"Without entering (says he) into the general consideration of these disorders, I shall only take notice of such cases as I have found most frequent in the army."

"Coughs and consumptions are properly annexed to the *inflammatory diseases*. For a recent cough, from cold, may be considered as the *lowest degree* of a *peripneumony*, and an old *neglected cough* as the *beginning* of a *consumption*.

*Obstructions* of the lungs are succeeded by tubercles, which making the cough worse, at last tear and ulcerate that organ. In all the bodies which I dissected, of those who died of the phthisis pulmonalis, I found the lungs full both of tubercles and ulcers. We cannot, therefore, be too careful in removing colds in the beginning."

"The disease being of an inflammatory nature, *bleeding* is the chief remedy; which, alone, will often cure bad colds, whilst all other medicines may be ineffectual without it."

"In the older and more stubborn coughs, *or in the first stage of a consumption*, when the patient complains of pains in his side, constriction at the breast, or hot and restless nights, I have trusted most to *small* but *repeated bleedings*, to issues, and to a low and cool diet.

I have found these *small bleedings* not only beneficial in old coughs, threatening consumptions, but also after hectic symptoms had appeared. The quantity of blood drawn was from *four* to *seven* or *eight* ounces once in eight or ten days; and sometimes a vein was opened after shorter intervals. It was observable that the patients never found themselves so much relieved on the first, as on the second or third night after bleeding. The blood was constantly sizy; but if it had ever been in a resolved state, to have insisted upon taking away more would have been altogether improper. Nor would I recommend this method for common practice, without making great allowance for the strength of soldiers, and *suiting the quantity of blood to be let, to the condition* of weaker patients."

Old MORTON, as Dr. Malden observes, has made nearly similar remarks.

"Although bleeding (says he) may do mischief sometimes in a confirmed consumption, yet, in the beginning of one, it is very beneficial: and I do not at all doubt but the *tubercles*, for the most part, *are occasioned by the neglect of it*, or for want of bleeding to a sufficient quantity *in the beginning of the distemper*, whereby the consumption uses to run presently into the second and more fatal degre.

"For so long as the lungs are only *stuffed*, or the *tubercles* that arise from that *stuffing* remain in a crude state, the person's life, though it be miserable, yet is not brought into any sudden danger, though he is troubled with an oppression of his chest, some difficulty of breathing, and a frequent cough."*

Dr. Malden argues strenuously and ingeniously in favour of the doctrine, which is now very generally admitted, that, whatever be the nature and origin of pulmonary tubercles, inflammatory affections of the lungs hasten their progress, and give strength to their fatality.

Among the exciting causes, catarrh, bronchitis, measles, hooping-cough, pleurisy, carditis, &c. are the most common; and the connexion between scrofula and phthisis leads our author to draw a parallel between the two diseases, which we shall give in his own words.

"Now, if upon inquiry it shall be found that, in degenerations (in other parts than the lungs,) admitted to be scrofulous, the process commences by a loaded state of the vessels of the affected part, and that this state is followed by an interstitial deposit of tubercles, then I think it will be fair to infer that the process in the formation of tubercles in the lungs is similar.

I have examined, with great care, the external lymphatic glands, and the mesenteric glands, (two structures which may be considered peculiarly liable to scrofulous disease,) in the various stages of this complaint, and have no hesitation in stating that the following is the course it pursues when it attacks these glands:—

A gradual enlargement takes place; this is sometimes very slow, at other times faster. For some time after the commencement of this enlargement of the gland, it is found, when cut into, to have a preternatural *pinkness*, (there is no other word that will express the peculiar colour); this pinkness, when it is examined with a magnifying glass, is found to be owing to innumerable small blood-vessels. At the same time that you meet with glands which have only this appearance, you will find others, in the same subject, which, upon close inspection, have, conjoined with the pinkness, small scattered white points in them, not larger than the smallest pins' heads. These little points picked out, are found of a cheesy consistence, and are, in all respects, similar to the tubercle of the lungs, in its first stage of softening.

If the disease be farther advanced,

* "Phthisiologia, p. 119."

then the quantity of this *steatoid* matter is increased; the gland assumes an irregular botryoidal figure; and when cut into, no longer exhibits the pink appearance before described, but contains small roundish masses of a whitish soft pulp, closely packed together, and divided from each other by delicate membranous bands.

As the disease goes on, these masses soften in the centre, and, gradually coalescing, form at last, a cavity in the gland, which contains a thin serum, (sometimes of a pink colour,) with which is mixed a soft curdy matter, and sometimes pus.

In the tabes mesenterica, children sometimes die of marasmus, in the first stage of the disease above described, and before the tubercular process has commenced.—Burns, in his chapter on this disease, describing the enlarged glands, expressly states that 'these tend slowly to the formation of a cheesy substance, but death may take place before that process be accomplished.' —*Burns' Midwifery*, p. 452.

The high vascularity of the ends of the bones, particularly of the cancellar structure, in the 'joint evil,' is familiar to all acquainted with this subject; and this increased vascularity is sometimes found with, and sometimes without, an interstitial deposit of tubercular matter.

I need not prosecute this part of my subject farther, since I have advanced enough to shew the great similarity, if not identity, of the morbid processes in scrofula, and tubercular 'phthisis:' and, notwithstanding all that has been adduced upon the opposite side of the question, I must confess that I think I am borne out by facts, when I state that a preternatural fulness of the blood-vessels of the part, precedes the tubercular degeneration in both instances. Whether this fulness be necessarily an adjunct of inflammation, I leave to others to decide."

It is evident that though living in the same atmosphere, and even drawing their public experience from the same institution, Drs. Baron and Malden have come to opposite conclusions respecting the pathology of tubercles. At this, however, we need not wonder. A word or two respecting the treatment of phthisis. Dr. Malden considers the following to be the most rational — namely, to equalize, by all the means in our power, the pulmonic and general circulation.

"These means are, — 1st. The subtraction of small quantities of blood from the *venous* system at short intervals, in proportion to the strength and age of the patient, and other circumstances. — 2dly. The maintenance, by warm clothing and other means, of a vigorous circulation in the *systemic* capillaries; and in particular, in those of the surface of the body (especially of the chest) and in those of the extremities. — 3dly. The preservation of the due nutrition of the body, by maintaining a healthy action of the whole digestive apparatus, and giving, at proper intervals, bland nutritious food.—4thly. The avoiding of all the acknowledged lædentia in the inspired air. — 5thly. The substituting, for walking exercise, exercise which does not produce much muscular pressure on the *valvular veins*, as this latter kind of exercise much assists the equalization of the circulation, whereas the former, as long as the lungs are oppressed, increases the mischief, by returning the blood to the right cavities of the heart faster than the lungs can bear it with convenience. Sydenham, who was a plain matter-of-fact man, would not have praised horse exercise in this disease so highly, if he had not found wonderful good effects from it.*—Lastly. Establishing sufficient drains from the parietes of the chest, by means varied according to the circumstances of the case. The ancients, although bad pathologists, as far as the light of morbid anatomy is concerned, were, on the whole, very fair practitioners; and the freedom with which they used the actual cautery in this disease, is worthy of remark.

'Exulcerandum est ferro candenti,

---

* "I solemnly affirm that riding is as effectual a remedy in this disorder, as mercury is in the lues venerea, or the bark in intermittens, &c.—*Swan's Sydenham*, p. 402.

uno loco sub mento, altero in gutture, duobus ad mammam utramque; item sub imis ossibus scapularum, quas *ομοπλατας* Græci vocant, *sic, ne sanescere ulcera sinamus, nisi tussi finitâ.*'—(Celsus lib. iii. cap. 22.)"

